# Proteomic mapping of Rosa damascena nanovesicles reveals plastid mitochondrial metabolic convergence and antimicrobial peptides

**DOI:** 10.1080/15592324.2026.2641285

**Published:** 2026-03-09

**Authors:** Subhashini Brahadeeswaran, Ramasamy Tamizhselvi

**Affiliations:** a School of Biosciences and Technology, Vellore Institute of Technology, Vellore, India

**Keywords:** Antimicrobial peptides, chloroplast, metabolism, mitochondria, nanovesicles, proteomics, rosa damascena

## Abstract

*Rosa damascena* exhibits diverse biological activities, including antimicrobial, antioxidant, anti-inflammatory, cardioprotective, neuroprotective, and skin-protective effects, largely attributed through its rich phytochemical composition. In parallel, plant-derived nanovesicles (PD-NVs) have emerged as natural nanocarriers that transport bioactive cargos capable of modulating recipient cell functions across kingdoms. In this study, *Rosa damascena* derived nanovesicles (RD-NVs) were isolated by ultracentrifugation and characterized by Transmission electron microscopy, nanoparticle tracking analysis, zeta potential measurement, and SDS-PAGE to confirm their vesicular nature, size distribution, and protein cargo profile. LC‒MS/MS-based proteomics, followed by annotation against NCBI and UniProt, and comparative BLASTP mapping to plant and human proteins, revealed 75 proteins shared with plant, *Arabidopsis* and human orthologs, which were further analyzed using interaction networks and hub detection together with GO and KEGG enrichment. RD-NVs (size, 40–100 nm; surface charge, −25 to −40 mV) carry proteins involved mainly in plastid transcription, ribosomal function, and photosynthetic electron transport in plants, whereas human mapped orthologs were enriched in oxidative phosphorylation, mitochondrial function, arginine-proline, taurine, and hypotaurine metabolism, suggesting that RD-NV hub proteins may interfere with metabolic pathways associated with obesity, insulin resistance, fatty liver, type 2 diabetes, and related inflammatory and neuroinflammatory disorders. Moreover, the peptides of the RD-NVs revealed similarities with multiple reported antimicrobial peptides therefore, might actively participate in plant and human defense system. Overall, this study reveals that the RD-NV proteome contains conserved pathways that may support metabolic and immune-related functions in cross-kingdom contexts. To validate cross-kingdom interaction *in vitro*, the RD-NVs were treated with the RAW264.7 macrophage which showed significant biocompatibility with a particle range of up to 8.94 × 10^8^ and cellular internalization.

## Introduction

In recent years, plant-derived nanovesicles (PD-NVs) have been widely recognized as promising therapeutic treatment strategy^1,2^ owing to their intrinsic therapeutic properties such as antimicrobial, anti-inflammatory, antioxidant, and anticancer effects, as well as their capability to serve as effective drug delivery system. As dietary components, PD-NVs can remain stable in gastrointestinal environment and taken up by intestinal and systemic cells to deliver bioactive cargos such as proteins, peptides, lipids, and nucleic acids, thereby reshaping host metabolism and immune responses.^1^

Presently, nanovesicles (NVs) derived from many edible plants such as ginger, cabbage, tea flowers, and grapes carry diverse cargo of proteins, RNA, lipids, metabolites that exert protective effects in metabolic disorders, cardiovascular disease, neurodegenerative diseases, and cancer.^3‐7^ Most recent studies have focused on decoding their RNA molecules, however, their mechanistic actions perhaps, because of the proteins rather than RNAs, due to their high biocompatibility, low immunogenicity, and biochemical response.^8^ By leveraging high-resolution LC-MS/MS, researchers have sorted the NV proteins and peptides, and revealed its regulatory role on oxidative stress, inflammation, apoptosis, and metabolic disorders.^9^ While PD-NVs are also known to regulate plant intercellular communications, cellular signaling, and host-pathogen defense system.^10^ Moreover, PD-NVs proteins contributes to plant defense mechanism.^11^ Besides, EVs also recognized as potential biomarker for the inflammatory diseases and cancer owing to the presence of key proteins involved in protein synthesis, oxidative response, phagosome, and ribosomal pathway.^12^

Proteomic analyzes of PD-NVs have been conducted for hardly any plant species,^13^ and rose flower (*Rosa damascena*) is not among this, despite being widely used in traditional medicine and known for its antioxidant, anti-inflammatory, antiseptic,^14^ and antipsychotic effect.^15‐17^ Therefore, further research is imperative to unveil the biologically active molecules present in rose flower, thus unlocking their potential in therapeutic applications. The present study systematically profiled the proteomic cargo of flower, *Rosa damascena* derived nanovesicles (RD-NVs) using LC-MS/MS, and bioinformatic analyzes. The functional roles of the proteins are interrogated in both plant and human signaling pathways including those linked to inflammation, oxidative stress, and cell death through gene ontology, pathway enrichment and network analyzes. Together, this work aims to delineate the direct and indirect molecular targets of exosome-like RD-NV proteins in the plant and human system and to position these vesicles as a candidate nanoplatform for future theragnostic applications. *In vitro* validation is essential for assessing biocompatibility and cellular uptake of plant-derived NVs. Macrophages represent a relevant immune cell model for cross-kingdom signaling given their roles in inflammation, phagocytosis, and metabolic regulation. Therefore, we evaluated cytotoxicity and cellular internalization of RD-NVs in RAW264.7 macrophages as a first step toward understanding mammalian interactions.

## Methods

### Isolation of RD-EVs from petals of *Rosa damascena*

Fresh *R. damascena* flowers were obtained from the local flower market in Vellore, India (12.934968°N, 79.146881°E). The petals were washed thoroughly with Milli-Q water three times to remove surface contaminants. Flowers were then soaked in ice-cold 1× PBS and homogenized using a kitchen blender in pulse mode (three cycles of 1 min each). The homogenate was subjected to sequential centrifugation at 1000 × g for 10 min, 3000 × g for 20 min, and 10,000 × g for 40 min at 4 °C to remove large debris and unwanted particulates. The clarified supernatant was ultracentrifuged at 150,000 × g (42,000 rpm) for 90 min at 4 °C (using an MLA-50 fixed angle rotor, Beckman Coulter), and the resulting pellet containing RD-NVs was resuspended in 1× PBS.^18^ The purified RD-NVs were stored at −80 °C until further experimental use.

### Transmission electron microscopy (TEM)

To visualize the morphology of the RD-NVs structure, TEM was performed using gold/carbon-coated copper meshes and phosphotungstic acid counterstaining, as previously described.^19^ Since nanovesicles are nanosized, electron microscopy is a suitable high-resolution technique for their detection.

### Nanoparticle tracking analysis (NTA)

The concentration, hydrodynamic diameter, and zeta potential of RD-NVs were determined using fluorescent nanoparticle tracking analysis (F-NTA) (NanoSight NS300, Malvern Instruments). Purified RD-NVs were labeled with DiO fluorescent dye (2 µg/mL; 100 µM) for 30 min at room temperature in the dark. The labeled RD-NVs were ultracentrifuged at 150,000 × g for 90 min at 4 °C to separate dye-bound vesicles from unbound dye. The pellet was washed with ice-cold PBS and ultracentrifuged again under the same conditions to ensure the removal of excess dye before the final resuspension in 1× PBS. For F-NTA measurements, RD-NV samples were diluted 1:100 in PBS and introduced into the NTA sample chamber (1 mL). The particle size and concentration were quantified under Brownian motion. Zeta potential analysis was also performed using the same instrument to assess surface charge and colloidal stability.

### BCA protein quantification and SDS-PAGE

Total RD-NVs protein concentration was measured using a bicinchoninic acid (BCA) assay (Takara, Japan). RD-NVs were mixed with BCA working reagent and incubated at 37 °C for 30 min in the dark. The absorbance was measured at 562 nm, and the protein concentration was calculated from a standard curve. For protein profiling, RD-NVs were lysed in RIPA buffer, mixed with SDS Laemmli buffer containing *β*-mercaptoethanol, glycerol, and loading dye, and heated at 95 °C for 10 min. Proteins were separated on 12% SDS-PAGE (5% stacking gel) at 50 V (stacking) and then 100 V (resolving). The gels were stained with Coomassie Brilliant Blue to visualize the protein bands, and the molecular weights were compared with protein ladder (HiMedia).

### LC–MS/MS analysis

RD-NV protein extracts were sonicated for 1 min to release intravesicular proteins, and ~50 µg of protein was denatured in 7 M urea (100 µL). Disulfide bonds were reduced using 50 mM tris (2-carboxyethyl) phosphine (TCEP) at 37 °C for 1 h and alkylated with 50 mM iodoacetamide (IAA) for 30  min at 37 °C. The samples (RD-NVs) were diluted with 25 mM NH₄HCO₃ to reduce the urea concentration and digested with trypsin at a 1:30 enzyme-to-substrate ratio for 16 h at 37 °C. The peptides were dried using a speed vacuum and reconstituted in 0.1% formic acid. Desalting was performed using a Thermo Scientific C18 Pierce column. Columns were activated with 50% methanol/50% acetonitrile (ACN), equilibrated with 5% ACN/0.5% TFA, loaded with peptide samples, washed, and eluted with 70% ACN. Processed RD-NV samples were vacuum-dried and stored at −80 °C. For LC-MS/MS, mobile phase A was 0.1% formic acid in water, and mobile phase B was 85:15 ACN:water with 0.1% formic acid.

Peptide separation was performed using a Thermo EASY-nLC system coupled to a Q Exactive Plus Orbitrap mass spectrometer (Thermo Scientific). The samples (12 µL injection volume) were loaded onto a precolumn (Acclaim PepMap 100, 100 µm × 2 cm) and separated on an analytical column (PepMap RSLC C18, 2 µm, 100 Å, 50 cm). Chromatographic separation was carried out at a constant flow rate of 300 nL/min using solvent A (0.1% formic acid in Milli-Q water) and solvent B (85:15 acetonitrile:Milli-Q water containing 0.1% formic acid). The gradient was programmed as follows: 2%–5% B (0–5 min), 5%–15% B (5–55 min), 15%–45% B (55–75 min), and 45%–95% B (75–85 min), followed by column washing at 95% B (85–90 min). The total run time was 90 min. The column was operated under ambient laboratory temperature conditions. Mass spectrometric analysis was performed in positive ion mode using a data-dependent acquisition method (full MS/dd-MS², Top15). Full MS scans were acquired at a resolution of 70,000 over an m/z range of 350–2000 with an AGC target of 1 × 10⁶ and a maximum injection time of 60 ms. MS² scans were acquired at a resolution of 17,500 with an AGC target of 2 × 10⁵, a maximum injection time of 120 ms, an isolation window of 1.2 m/z, and a normalized collision energy (NCE) of 27. Dynamic exclusion was set to 15 s, and singly charged and highly charged ions (>7) were excluded from fragmentation. The analysis was performed using two independent biological replicates. RAW files were converted to mzML using ThermoRawFileParser (1.4.5) and to mzid using MS-GF+ (2024.03.26), which were matched against *R. damascena* protein sequences. Confident proteins were identified using PeptideShaker (v3.0.2). Protein identification thresholds were filtered at 1% peptide- and protein-level FDRs. Missing values were not artificially imputed. Relative label-free quantification was performed in PeptideShaker using normalized spectral abundance metrics based on total spectrum counts under default parameters. For peptide sequence extraction, mzid files were converted to OpenMS-compatible idXML format using OpenMS tools. Peptide sequences were then extracted and exported in FASTA format for downstream analysis and antimicrobial peptide screening. Only high-confidence protein identifications were considered for further analysis.

BLASTP was used to identify sequence-similarity matches for the RD-NV proteins against *Arabidopsis thaliana* (TAIR10) and *Homo sapiens* (UniProt) proteomes. To evaluate the antimicrobial potential of the detected peptides, a local BLAST database was generated from the dbAMP3 antimicrobial peptide repository (dbAMP server), and peptide FASTA files obtained from the mzid output were queried against the dbAMP3 database using BLASTP short with default parameters. High-confidence antimicrobial peptide (AMP) candidates were defined as those exhibiting ≥90% sequence identity to known AMPs in dbAMP3. Matching AMP entries were further subjected to downstream annotation and functional interpretation.

### Functional and pathway enrichment analysis

Gene Ontology (GO) and Kyoto Encyclopedia of Genes and Genomes (KEGG) pathway analyzes were performed using ShinyGO (v0.85) webserver to characterize the biological functions and signaling pathways represented in the RD-NV proteome.

### Protein‒protein interaction network analysis

Protein‒protein interaction networks (PPI) were generated using STRING (https://string-db.org/) by uploading all identified proteins in “Multiple proteins” mode. The networks were visualized in Cytoscape, and hub proteins were ranked using the cytoHubba plugin. Topological algorithms, including closeness, degree, maximum neighborhood component (MNC), and maximal clique centrality (MCC), were applied to identify the most functionally significant nodes. Venn diagram analysis was conducted where appropriate.

### Cell culture maintenance

RAW264.7 murine macrophage cells (National Center for Cell Sciences, Pune, India) were cultured in DMEM supplemented with 10% fetal bovine serum and 1% antibiotic solution at 37 °C in a humidified atmosphere containing 5% CO_2_. The cells were maintained in T25 flasks and passaged upon reaching approximately 80% confluency. For subculture, the cells were mechanically detached by scraping in fresh DMEM, collected in Falcon tubes, and centrifuged at 1500 rpm for 5 min. The supernatant was discarded, and the cell pellet was resuspended in DMEM supplemented with 10% FBS. A consistent split ratio of 1:4 was used to control the passage number throughout the experiments. Cell counts were determined using a hemocytometer.

### Cell viability assay

The cytotoxicity of RD-NVs was evaluated using the MTT assay.^20^ RAW264.7 murine macrophages were seeded in 96-well plates and treated with increasing concentrations of RD-NVs (0, 8.94 × 10^7^, 1.78 × 10^8^, 2.68 × 10^8^, 4.47 × 10^8^, 8.94 × 10^8^, 2.23 × 10^9^, 2.68 × 10^9^, 4.02 × 10^9^, and 4.47 × 10^9^ particles) suspended in 1× PBS. After 24 h of incubation at 37 °C, 10 µL of MTT reagent (5 mg/ml) was added to each well to assess cell viability via formazan crystal formation. Formazan crystals were subsequently solubilized with 100 µL of DMSO, and the absorbance was recorded at 570 nm to quantify viable cells.

### Cellular uptake – fluorescence microscopy imaging

Visualization of DiO-labeled RD-NVs and their internalization by RAW264.7 macrophages was performed using an EVOS M5000 Imaging System. The dynamic uptake of RD-NVs was monitored through live-cell imaging. The macrophages were incubated with 5 µL of DiO-stained RD-NVs, and internalization was documented after 30 min at 20× magnification with a scale bar of 150 µm.

#### Statistical analysis

Statistical analysis for the MTT assay was performed using GraphPad Prism (version 8). Data are presented as mean ± standard deviation (SD) from three independent experiments. Comparisons among multiple groups were performed using one-way ANOVA followed by Tukey's multiple comparison test. A *p*-value <0.05 was considered statistically significant. Gene Ontology (GO) and Kyoto Encyclopedia of Genes and Genomes (KEGG) pathway enrichment analyzes were performed using over-representation analysis based on hypergeometric testing implemented in the ShinyGO platform. *P*-values were adjusted using the Benjamini‒Hochberg false discovery rate (FDR) correction method. Adjusted *p*-values (FDR < 0.05) were considered statistically significant.

## Results

### Morphological and physicochemical characteristics of RD-NVs

**Figure 1.**
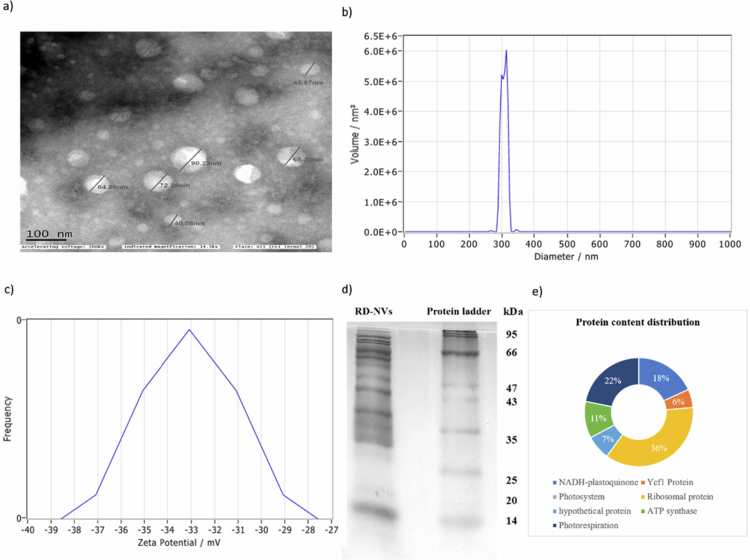
Physical and biological characterization of isolated RD-NVs. (a) Transmission electron microscopy (TEM) visualized NVs as spherical vesicles with diameters ranging from ~40 to 100 nm. (b) Nanoparticle tracking analysis (NTA) indicated a particle size distribution predominantly between 250 and 300 nm. (c) Zeta potential measurements by NTA revealed that NVs possess a surface charge of approximately −33 mV. (d) SDS-PAGE (12%) showed protein cargo spanning ~14–100 kDa. (e) The relative abundance of NV-associated proteins is illustrated as percentages in a donut pie chart.

Initial characterization confirmed the successful isolation of RD-NVs. TEM imaging revealed uniformly spherical vesicles with smooth surfaces and diameters ranging between approximately 40 and 100 nm (Figure 1a), which is consistent with the expected size of plant-derived nanovesicles. NTA further validated this observation, showing a monodisperse population with a peak size distribution centered around 250–300 nm (Figure 1b) and high particle counts within this range. These measurements confirm that the isolated particles fall within the characteristic size window reported for plant NVs.

Zeta potential analysis demonstrated that the vesicles carried a negative surface charge, with values distributed between −40 and −27 mV and a peak around −33 mV (Figure 1c). This high negative zeta potential is typical of PD-NVs and is indicative of colloidal stability, suggesting that the vesicles maintain dispersion in aqueous environments and exhibit a low aggregation tendency. The larger hydrodynamic diameter observed in NTA relative to TEM is expected due to vesicle hydration shell and Brownian motion-based measurement principle; similar discrepancies are widely reported for plant NVs.

### Protein profiling of RD-NVs

Protein profiling by SDS-PAGE revealed a complex mixture of protein cargo, with multiple bands spanning a broad molecular weight range (Figure 1d). Prominent bands were visible between ~20–66 kDa, indicating the presence of diverse structural, enzymatic, and metabolic proteins typically associated with RD-NVs. This heterogeneous protein content supports subsequent proteomic and functional annotation analysis (Figure 1e).

**Figure 2.**
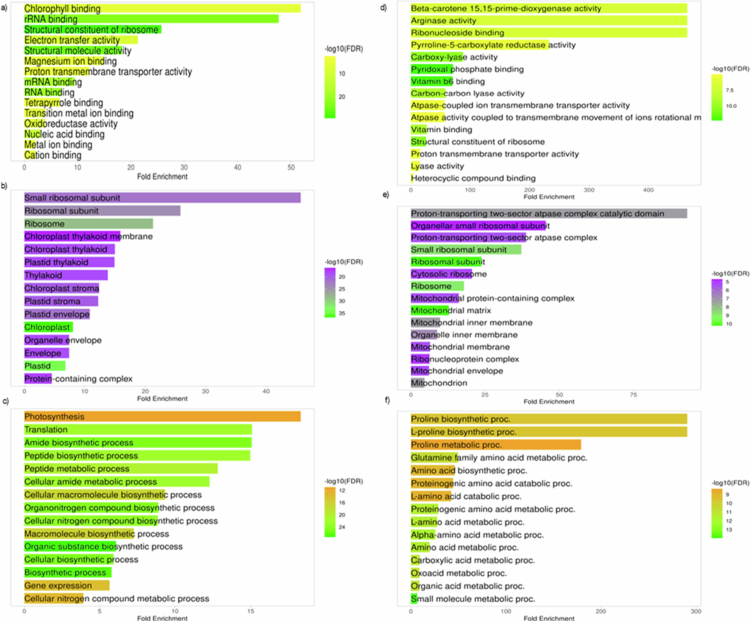
Gene ontology (GO) enrichment analysis of RD-NVs. Each bar plot displays significantly enriched categories across the molecular function (MF), cellular component (CC), and biological process (BP). (a–c) depicts the molecular function, cellular compartment, and biological process of the plant background gene (*Arabidopsis thaliana*), and (d–f) shows the molecular function, cellular compartment, and biological process in the human background gene (*Homo sapiens*). The horizontal bar length indicates Fold Enrichment, while the color gradient denotes −log10(FDR) significance levels, with darker color corresponding to more significant GO terms. Enrichment was performed using hypergeometric over-representation analysis in ShinyGO, and GO terms with adjusted FDR < 0.05 (Benjamini–Hochberg correction) were considered statistically significant.

Proteomics by LCMS/MS revealed presence of 75 *Rosa damascena* proteins with confidence which were mentioned in Table 1. The data revealed that RD-NV proteins clustered into distinct functional groups, with ribosomal proteins comprising the major proportion (36%), indicating a strong representation of the translational machinery within NVs. Importantly, chloroplast NAD(P)H dehydrogenase complex subunits involved in photosynthetic electron transport related proteins accounted for 18%, whereas photorespiration-related proteins constituted 22%, suggesting enrichment of components linked to chloroplast redox metabolism and carbon recycling.​ In addition, ATP synthase subunits (11%), hypothetical proteins (7%), and photosystem were also detected, consistent with a chloroplast origin of a subset of RD-NVs cargo.​ Moreover, 6% of Ycf1 (hypothetical chloroplast reading frame 1) chloroplast encoding protein and other hypothetical proteins, which essentially participate in the chloroplast biogenesis and membrane-associated proteins in specific NV components (Figure 2a).

**Table 1.** Representing the list of cargo proteins comprised in the RD-NVs.

Main accession	Description	MW [kDa]
UKP82244.1	hypothetical protein RF1 (chloroplast) [Rosa × damascena]	226.93
UKP82230.1	hypothetical protein RF2 (chloroplast) [Rosa × damascena]	267.84
UKP82217.1	RNA polymerase beta' subunit (chloroplast) [Rosa × damascena]	157.26
UKP82215.1	RNA polymerase beta subunit (chloroplast) [Rosa × damascena]	120.46
ABY47994.1	carotenoid cleavage dioxygenase 1 [Rosa × damascena]	62.33
QGW08886.1	linalool synthase protein, partial [Rosa × damascena]	64.19
QGW08887.1	geraniol synthase [Rosa × damascena]	68.62
UKP82205.1	ribosomal protein S4 (chloroplast) [Rosa × damascena]	23.37
AEX97877.1	maturase K (chloroplast) [Rosa × damascena]	59.66
ABY60886.1	carotenoid cleavage dioxygenase 4 [Rosa × damascena]	64.04
UKP82232.1	ribosomal protein S7 (chloroplast) [Rosa × damascena]	17.38
UKP82222.1	ATP synthase CF1 alpha subunit (chloroplast) [Rosa × damascena]	55.33
UKP82168.1	50S ribosomal protein L16 (chloroplast) [Rosa × damascena]	15.43
UKP82184.1	ribosomal protein L20 (chloroplast) [Rosa × damascena]	14.13
UKP82242.1	NADH-plastoquinone oxidoreductase subunit 7 (chloroplast) [Rosa × damascena]	45.71
UKP82218.1	ribosomal protein S2 (chloroplast) [Rosa × damascena]	26.92
UKP82207.1	photosystem I P700 apoprotein A1 (chloroplast) [Rosa × damascena]	83.00
QBK46548.1	AGAMOUS-like MADS-box, partial [Rosa × damascena]	27.08
QKV43327.1	mitochondrial arginase 1, partial [Rosa × damascena]	35.42
UKP82180.1	photosystem II CP47 chlorophyll apoprotein (chloroplast) [Rosa × damascena]	56.06
UKP82209.1	ribosomal protein S14 (chloroplast) [Rosa × damascena]	11.71
UKP82200.1	ATP synthase CF1 beta subunit (chloroplast) [Rosa × damascena]	52.75
UKP82203.1	NADH-plastoquinone oxidoreductase subunit K (chloroplast) [Rosa × damascena]	25.40
UKP82228.1	ribosomal protein L2 (chloroplast) [Rosa × damascena]	29.79
QGW08952.1	geranyl diphosphate synthase protein [Rosa × damascena]	41.54
UKP82170.1	ribosomal protein S8 (chloroplast) [Rosa × damascena]	15.48
UKP82185.1	ribosomal protein S18 (chloroplast) [Rosa × damascena]	12.01
QKV43323.1	delta-1-pyrroline-5-carboxylate synthase-like protein, partial [Rosa × damascena]	29.70
UKP82166.1	ribosomal protein L22 (chloroplast) [Rosa × damascena]	15.93
BAF64843.1	aromatic L-amino acid decarboxylase [Rosa × damascena]	56.48
UKP82233.1	NADH-plastoquinone oxidoreductase subunit 5 (chloroplast) [Rosa × damascena]	84.44
APT42958.1	geranylgeranyl pyrophosphate [Rosa × damascena]	39.50
BAG13450.2	phenylacetaldehyde reductase [Rosa × damascena]	35.36
AGO04401.1	1-deoxy-D-xylulose 5-phosphate reductoisomerase [Rosa × damascena]	51.28
UKP82173.1	ribosomal protein S11 (chloroplast) [Rosa × damascena]	15.04
UKP82221.1	ATP synthase CF0 subunit I (chloroplast) [Rosa × damascena]	20.93
UKP82196.1	photosystem I assembly protein Ycf4 (chloroplast) [Rosa × damascena]	21.34
UKP82229.1	ribosomal protein L23 (chloroplast) [Rosa × damascena]	10.71
UKP82243.1	ribosomal protein S15 (chloroplast) [Rosa × damascena]	10.85
UKP82167.1	ribosomal protein S3 (chloroplast) [Rosa × damascena]	25.18
UKP82194.1	cytochrome f (chloroplast) [Rosa × damascena]	34.98
UKP82182.1	ribosomal protein S12 (chloroplast) [Rosa × damascena]	13.66
UKP82186.1	ribosomal protein L33 (chloroplast) [Rosa × damascena]	7.67
QKV43318.1	pyrroline-5-carboxylate reductase-like protein, partial [Rosa × damascena]	24.09
UKP82208.1	photosystem I P700 apoprotein A2 (chloroplast) [Rosa × damascena]	82.32
UKP82195.1	envelope membrane protein (chloroplast) [Rosa × damascena]	27.17
UKP82225.1	ribosomal protein S16 (chloroplast) [Rosa × damascena]	10.40
UKP82211.1	photosystem II CP43 chlorophyll apoprotein (chloroplast) [Rosa × damascena]	51.82
UKP82181.1	clp protease proteolytic subunit (chloroplast) [Rosa × damascena]	22.00
UKP82165.1	ribosomal protein S19 (chloroplast) [Rosa × damascena]	10.56
UKP82198.1	acetyl-CoA carboxylase carboxyltransferase beta subunit (chloroplast) [Rosa × damascena]	54.79
WGU17157.1	clpP-like protease (plastid) [Rosa × damascena]	21.84
UKP82174.1	RNA polymerase alpha subunit (chloroplast) [Rosa × damascena]	38.24
ABV24888.1	GAI-like protein, partial [Rosa × damascena]	28.93
QKV43320.1	mitochondrial ornithine aminotransferase, partial [Rosa × damascena]	20.39
UKP82241.1	NADH-plastoquinone oxidoreductase subunit 1 (chloroplast) [Rosa × damascena]	40.05
CAA49408.1	ribulose-1, 5-bisphosphate-carboxylase, partial (chloroplast) [Rosa × damascena]	6.36
UKP82206.1	photosystem I assembly protein Ycf3 (chloroplast) [Rosa × damascena]	19.49
ABV24907.1	GAI-like protein, partial [Rosa × damascena]	28.95
QKV43321.1	delta-1-pyrroline-5-carboxylate synthase, partial [Rosa × damascena]	35.04
UKP82227.1	photosystem II protein D1 (chloroplast) [Rosa × damascena]	38.95
UKP82201.1	ATP synthase CF1 epsilon subunit (chloroplast) [Rosa × damascena]	14.62
UZS93161.1	glyceraldehyde 3-phosphate dehydrogenase, partial [Rosa × damascena]	11.26
UKP82169.1	ribosomal protein L14 (chloroplast) [Rosa × damascena]	13.58
UKP82177.1	photosystem II phosphoprotein (chloroplast) [Rosa × damascena]	7.84
UKP82235.1	cytochrome c heme attachment protein (chloroplast) [Rosa × damascena]	36.65
UKP82190.1	photosystem II cytochrome b559 alpha subunit (chloroplast) [Rosa × damascena]	9.38
UKP82216.1	RNA polymerase beta' subunit (chloroplast) [Rosa × damascena]	78.99
UKP82244.1	hypothetical protein RF1 (chloroplast) [Rosa × damascena]	226.93
AZZ85947.1	Ycf1 protein, partial (chloroplast) [Rosa × damascena]	32.83
AZZ85955.1	Ycf1 protein, partial (chloroplast) [Rosa × damascena]	20.91
AWM67312.1	ribulose-1, 5-bisphosphate carboxylase/oxygenase large subunit, partial (chloroplast) [Rosa × damascena]	50.60
AWM67345.1	ribulose-1, 5-bisphosphate carboxylase/oxygenase large subunit, partial (chloroplast) [Rosa × damascena]	51.77
AWM67313.1	ribulose-1, 5-bisphosphate carboxylase/oxygenase large subunit, partial (chloroplast) [Rosa × damascena]	52.23

### Cross-species mapping

**Figure 3.**
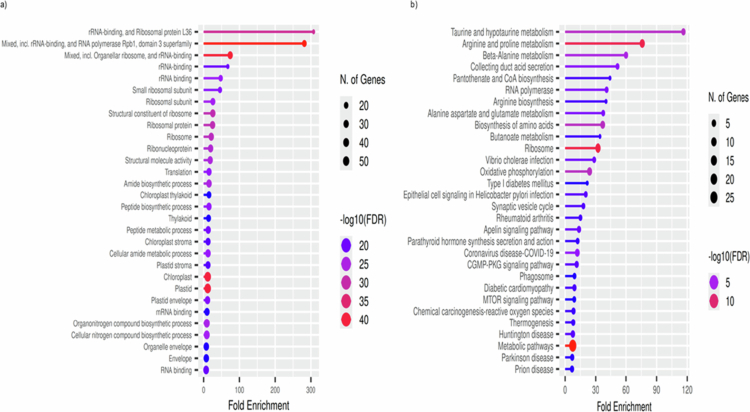
KEGG enrichment analysis of plant and human genes identified from *Rosa damascena* NVs. (a) Fold enrichment values are plotted along the x-axis, highlighting pathways related to ribosomal function, chloroplast activity, amino acid biosynthesis, and metabolic processes. (b) Fold enrichment values are plotted along the x-axis, highlighting pathways related to metabolic pathways in association with human-related genes. The bubble plot shows significantly enriched KEGG pathways associated with plant-derived genes present in the NV protein dataset. The bubble size indicates the number of genes, while bubble color represents the −log10(FDR) significance level. Enrichment analysis was performed using hypergeometric over-representation testing implemented in ShinyGO, and pathways with an adjusted FDR < 0.05 (Benjamini–Hochberg correction) were considered statistically significant.

**Figure 4.**
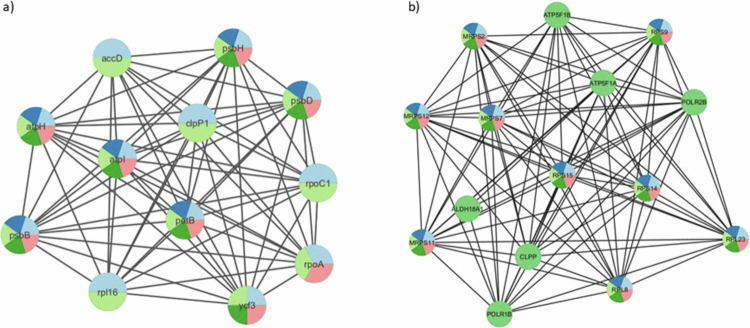
Protein‒protein interaction networks of the top-ranked hub proteins. a) shows the chloroplast-encoded plant hub proteins, and b) shows the energy and metabolic functional human hub proteins.

Proteomic profiling followed by cross-species similarity mapping identified a proteome enriched in evolutionarily conserved metabolic and regulatory pathways. Mapping to the *A. thaliana* background highlighted plastid-associated functions, while mapping to the human reference genome found to be involved in the mitochondrial and amino acid metabolic systems. Together, these analyses reveal a dual evolutionary signature reflecting both the plant origin of the vesicles and their potential functional relevance in mammalian cells. GO enrichment of *A. thaliana*-mapped proteins demonstrated a clear dominance of chloroplast-related processes (Figure 2a-c), with strong representation of pathways governing photosynthetic electron transport (e.g., psbA, psbD, psbC, psbH, psbB), carbon fixation (rbcL encodes the Rubisco large subunit in the Calvin cycle), plastid transcription‒translation coupling (rpoA, rpoC1, rpl16, rps12, rps3), redox buffering, and amino acid precursor biosynthesis (accD, DXR, GGPPS1). These functions are canonically associated with plant stress adaptation, where plastids serve as central hubs for reactive oxygen species (ROS) regulation and metabolic homeostasis (Figure 2b). KEGG enrichment further supported this profile, revealing pathways associated with photosystem assembly, Calvin cycle regulation, terpenoid backbone synthesis, and plastid proteostasis, including proteins such as CLPP4, CcsA, Ycf3, Ycf4, and ATP synthase-related genes (atpA, atpB, atpE). The coherence of these pathways indicates that RD-NVs carry an integrated plastid metabolic module rather than random chloroplast contaminants. This is reinforced by network analysis, which identified plastid gene expression and photosystem maintenance proteins such as psbA, psbD, rpoC1, accD, petB, and ribosomal constituents (rpl16, rpl14, rps11, rps18) as the highest-scoring hubs, suggesting that RD-NV proteins converge in maintaining redox stability, energy metabolism, and proteome activity (Figure 3a). Additionally, the CytoHubba network is highlighting a small group of chloroplast-related genes that behave as hubs in the interaction network built from RD-NV annotated proteins.​ CytoHubba ranked nodes by centrality using four different algorithms such as maximal clique centrality (MCC), maximum neighborhood component (MNC), degree, and betweenness; thus, each node shows its highest topological importance through protein‒protein interactions. As a result, clpP1, accD, psbH, psbD, rpoC1, rpoA, ycf3, petB, atpI, rpl16, and psbB are chloroplast-encoded proteins involved in photosynthesis, transcription/translation, or proteostasis, which are known to interact functionally and physically in chloroplast gene expression pathways (Figure 4a).​ Together, these results suggest RD-NVs are particularly enriched for chloroplast gene expression and photosystem-related proteins, and these hubs may be critical for maintaining or signaling chloroplast function in *A. thaliana* (plant).

In contrast to the plastid-derived patterns observed in the plant background, the human similarity analysis revealed a strong and unexpected convergence onto mitochondrial translation, oxidative phosphorylation, proton-coupled ATP synthesis, and amino-acid interconversion pathways. While the GO terms enriched in the human were found to participate in the pyridoxal phosphate (Vitamin B6) dependent catalysis, oxidoreductase activity, arginase activity, mitochondrial ribosome assembly, and ATPase-coupled proton transport (Figure 2d–f). KEGG pathways prominently featured arginine-proline metabolism, glutamate-derived nitrogen cycling, retinoid (Vitamin A) metabolism, and electron transport chain-associated processes. Notably, several RD-NV proteins mapped to human enzymes involved in retinal/retinoic acid production, including BCO1, BCO2, RPE65, and to proline biosynthesis, including PYCR1, PYCR2, PYCR3, as well as enzymes such as OAT, AGXT2, and ALDH18A1. These pathways play strong roles in epithelial repair, immune modulation, mitochondrial health, and collagen synthesis. The network analysis placed mitochondrial ribosomal proteins (MRPS2, MRPS7, MRPS11, MRPS12) and ATP synthase subunits (ATP5F1A, ATP5F1B, ATP6V1A, ATP6V1B1, ATP6V1B2) at the core of the human similarity network, suggesting that the RD-NV proteome is structurally aligned with essential nodes controlling cellular energy homeostasis, stress tolerance, and metabolic resilience (Figure 3b). Using CytoHubba network analysis, we identified hub proteins and their interactions among MRPS2, MRPS7, MRPS11, MRPS12, and related nodes that are majorly involved in mitochondrial small-subunit ribosomal proteins required for translation of mtDNA-encoded respiratory chain components and for maintaining mitochondrial energy metabolism.​ ATP5F1A/B and other ATP5 subunits are components of mitochondrial ATP synthase (complex V), which synthesizes ATP using the proton gradient across the inner mitochondrial membrane.​ RPL23, RPL8, RPS14, and RPS15 are cytosolic ribosomal proteins with roles in global translation and additional functions in cell cycle, p53 signaling, and stress responses.​ RNA polymerase subunits such as POLR2B and POLR1B association signify their linkage in mRNA and rRNA transcriptional activity. The dense edges indicate strong functional association among these proteins (co-expression, physical interaction, shared pathways). As a result, the CytoHubba report suggests that the core mitochondrial translation, oxidative phosphorylation, and ribosome module are involved as the central hub that can be targeted by RD-NV-annotated human proteins (Figure 4b).​

### Peptidome screening reveals antimicrobial peptides enriched in RD-NVs

Plant-derived antimicrobial peptides (AMPs) are integral to innate defense and include defensins, cyclotides, thionins, hevein-like peptides, and ribosomal fragments. These peptides possess cationic charge and amphiphilic surfaces that confer broad-spectrum antibacterial and antifungal activity. Emerging studies suggest that AMPs can be selectively packaged into PD-NVs, facilitating intercellular defense signaling. Based on this, we examined whether RD-NVs contain AMP-like peptides through peptidome analysis. Proteomics analysis of *R.* *damascena-*derived RD-NVs yielded 26 peptides with ≥90% sequence similarity to antimicrobial peptides (AMPs) in the dbAMP database, highlighting a rich repertoire of bioactive defense molecules (Table 2). The physicochemical properties of these peptides predominantly showed 66–99 residues long (average amino acid residues: 79.23), exhibited net charge, charge density and isoelectric point (average net charge: +10.5 and 11.3; pI 10.37) with moderate amphiphilicity index (average index: 0.96) (Table 3), thus matches with the hallmarks of broad-spectrum antimicrobial activity against fungal and bacterial pathogens. Further, seventeen peptides (65%) aligned with synthetic sequences from U.S. patents US7166769 and US6573361B1, including FCWP-family antifungals (IDs 04000, 04081-04085, 25900, 25955-25956, 26294-26298, 26340, 26428, 26549-26557) validated against *Fusarium culmorum, Botrytis cinerea, Alternaria brassicicola*, and *Rhizoctonia solani*. The remaining matched natural AMPs from *Pelargonium zonale* (Q6V0P7*), Fagopyrum spp.* (Q0VYL5), pepper, and Capsicum, with compact structures (e.g., ID 32554: 28 aa; ID 34428: 21 aa) suited for RD-NVs cargo. Also, the shorter sequences (IDs 32554, 34428) mirrored pepper/Capsicum defensins, underscoring cross-species conservation of EV-packaged AMPs. This dataset positions RD-NVs as potent carriers of metabolically stable, patent-validated antifungals with agricultural and therapeutic promise.

**Table 2.** Representation of antimicrobial peptide cargo identified in RD-NVs showing ≥90% sequence similarity.

S.No	dbAMP ID	Peptide Sequence	Sequence length	Name and reference ID
1	dbAMP_04000	GRLHPQDCQPKCTYRCSKTSYKKPCMFFCQKCCAKCLCVPAGTYGNKQSCPCYNNWKTKRGGPKCP	66	Synthetic Sequence 81 from patent (Patent ID: US 7166769)
2	dbAMP_04081	GSLKSSQCNPECTRRCSKTQYHKPCMFFCQKCCRKCLCVPPGFYGNKAVCPCYNNWKTQQGGPKCP	66	Q6V0P7_9ROSI Pelargonium zonale Gasa4-like protein (Predicted)
3	dbAMP_04083	GSLKSYQCPSQCSRRCSKTQYHKPCMFFCQKCCKKCLCVPPGYYGNKAVCPCYNNWKTKEGGPKCP	66	Q0VYL5_FAGSY (Fagus sylvatica)
4	dbAMP_04085	GSLQPQECGPRCSERCSNTQYKKPCLFFCNKCCAKCLCVPPGTYGNKQFCPCYNNWKTKRGGPKCP	66	Q5EDG3_9ROSI (UniprotKB ID: Q5EDG3) (Pelargonium zonale)
5	dbAMP_06218	MAGINKLSALLVVIALGYLLAPSTEGFVLSDCYDTWSRCSGWSSALTGILWNTCSERCQCLGHADGACHLAQTNCGEAYQCQCHGTLNGPRPSNCKF	97	20639135 (UniprotKB ID: D9YTA7) (PubMed ID: 20639135)
6	dbAMP_25900	GSLHPQDCQPKCTYRCSKTSFKKPCMFFCQKCCAKCLCVPAGTYGNKQTCPCYNNWKTKEGGPKCP	66	Synthetic Sequence 85 from patent (Patent ID: US 7166769)
7	dbAMP_25955	IFLLTLIVLFMLQTMVMASSGSNVKWRQKRYGPGSLKRTQCPSECDRRCKKTQYHKACITFCNKCCRKCLCVPPGYYGNKQVCSCYNNWKTQEGGPKCP	99	Synthetic Sequence 79 from patent (Patent ID: US 7166769)
8	dbAMP_25956	IFLLTLIVLFMLQTMVMASSGSNVKWSQKRYGPGSLKRTQCPSECDRRCKKTQYHKACITFCNKCCRKCLCVPPGYYGNKQVCSCYNNWKTQEGGPKCP	99	Synthetic Sequence 78 from patent (Patent ID: US 7166769)
9	dbAMP_26294	MARSLKKAPFVANHLLEKVERLNTQGDKKVIKTWSRSSTIVPLMIGHTIAVHNGREHIPVFITDQMVGHKLGEFAPTRTFRGHVKKDKKSKR	92	Synthetic Sequence 1210 from patent (Patent ID: US 6573361)
10	dbAMP_26298	MARSLKKNPFVANHSLRKIKNLNIKEEKKIIVTWSRASVIVPAMIGHTIAVHNGREHLPIYVTDRMVDHKLGEFAPTLLFQGHARNDKKSRR	92	Synthetic Sequence 1219 from patent (Patent ID: US 6573361)
11	dbAMP_26340	MIHSPTLKKNLFVANHLRAKINKLNNKKKKEIIVTWSRASTIIPIMIGHMISIHNGKEHLPIYITDHMVGHKLGEFVPTLNFRGHAKSDNRSRR	94	Synthetic Sequence 1212 from patent (Patent ID: US 6573361)
12	dbAMP_26428	MLKLRLKRCGRKQRFYDPIKNQTCLNVPAILYFLEKGAQPTRTVSDILRKAEFFKEKERTLS	62	Synthetic Sequence 1189 from patent (Patent ID: US 6573361)
13	dbAMP_26549	MVKLRLKRCGRKQQAIYRIVAIDVRSRREGRDLRKVGFYDPIKNQTCLNVPAILYFLEKGAQPTRTVYDILRKAEFFKDKERTLS	85	Synthetic Sequence 1187 from patent (Patent ID: US 6573361)
14	dbAMP_26550	MVKLRLKRCGRKQQAVYRIVAIDVRSRREGRDLRKVGFYDPIKNQTCLNVPAILYFLEKGAQPTRTVYDILRKAEFFKEKERTLS	85	Synthetic Sequence 1186 from patent (Patent ID: US 6573361)
15	dbAMP_26551	MVKLRLKRCGRKQRAVYRIVAIDVRSRREGKDLQKVGFYDPIKNQTYLNVPAILYFLEKGAQPTETVQDILKKAEVFKELRLNQPKFN	88	Synthetic Sequence 1192 from patent (Patent ID: US 6573361)
16	dbAMP_26552	MVKLRLKRCGRKQRAVYRIVAIDVRSRREGKDLRKVGFYDPIKNQTYLNVPAILYFLEKGAQPTGTVQDILKKAEVFKELRPNQS	85	Synthetic Sequence 1193 from patent (Patent ID: US 6573361)
17	dbAMP_26553	MVKLRLKRCGRKQRAVYRIVAIDVRSRREGRDLRKVGFYDPITNQTYLNLPAILDFLKKGAQPTRTVHDISKKAGIFTELNLNKTKLN	88	Synthetic Sequence 1191 from patent (Patent ID: US 6573361)
18	dbAMP_26554	MVKLRLKRYGRKGQVTYRIVAMNNLSRRDGKAIEELGFYNPRTNESSLNIANIKRRIEQGAQPTNTVRYILAKANIL	77	Synthetic Sequence 1185 from patent (Patent ID: US 6573361)
19	dbAMP_26555	MVKLRLKRYGRKQQPSYRIVAMDSRSKRDGKAIEELGFYNPITNETRIDIAKILKRLKQGAQTTRTVKNILNEAQIIAKENS	82	Synthetic Sequence 1190 from patent (Patent ID: US 6573361)
20	dbAMP_26567	MYKFKRSFRRRLSPIGSGNLIYYRNMSLISRFISEQGKILSRRVNRLTLKQQRLITIAIKQARILSLLPFINNEKQFERIESITRVKGFIKK	92	Synthetic Sequence 1200 from patent (Patent ID: US 6573361)
21	dbAMP_26947	TRSLKKNPFVANHLLKKIDKLNTKAEKEIIVTWSRASTIIPTMIGHTIAIHNGKEHLPIYITDSMVGHKLGEFAPTLNFRGHAKSDNRSRR	91	Synthetic Sequence 1224 from patent (Patent ID: US 6573361)
22	dbAMP_26948	TRSLKKNPFVANHLLRKIEKLNKKAEKEIIVTWSRASTIIPTMIGHTIAIHNGREHLPIYITDRMVGHKLGEFAPTLNFRGHAKNDNKSRR	91	Synthetic Sequence 1223 from patent (Patent ID: US 6573361)
23	dbAMP_26949	TRSLKKNPFVANHLLRKINKLNTKAEKDIIITWSRASTIIPTMIGHTIAIHNGKEHLPIYITDRMVGHKLGEFSPTLNFRGHAKNDNRSRR	91	Synthetic Sequence 1222 from patent (Patent ID: US 6573361)
24	dbAMP_26950	TRSRKKNPFVANHLLKKIKKLNTKGEKAIIKTWSRKSTIIPIMIGHTIAIHNGKEHLPVYITDRMVGHKLGEFSPTLNFGGFAKNDNKSRR	91	Synthetic Sequence 1218 from patent (Patent ID: US 6573361)
25	dbAMP_32554	NIIPSSTGAAKAVGKVLPALNGKLTGMA	28	NP-1 Zanthoxylum bungeanum (Sichuan pepper) (PubMed ID: 30623204)
26	dbAMP_34428	DWRGGRTASGNIIPSSTGAAK	21	Peptide 20 Saccharomyces cerevisiae (yeast) (PubMed ID: 35215278)

**Table 3.** Representing the physicochemical properties of the antimicrobial peptides in RD-NVs.

S.No	dbAMP ID	Molecular weight	Net charge	Isoelectric point	Charge density	Amphiphilicity index
1	dbAMP_04000	7475.85	12.00	9.18	12.28	1.18
2	dbAMP_04081	7473.79	10.00	8.75	10.28	1.03
3	dbAMP_04083	7496.87	11.00	8.93	11.27	1.24
4	dbAMP_04085	7401.66	9.00	8.57	9.18	1.01
5	dbAMP_06218	10361.8	−1.00	6.27	−0.38	0.66
6	dbAMP_25900	7377.7	9.00	8.57	9.28	1.05
7	dbAMP_25955	11356.5	14.00	9.56	14.28	1.07
8	dbAMP_25956	11287.4	13.00	9.36	13.28	1.04
9	dbAMP_26294	10481.3	12.00	11.35	13.59	0.92
10	dbAMP_26298	10576.4	11.00	11.28	12.59	0.91
11	dbAMP_26340	10840.8	12.00	11.36	13.78	0.88
12	dbAMP_26428	7388.77	9.00	10.70	9.864	1.06
13	dbAMP_26549	10089.9	12.00	10.79	12.86	1.09
14	dbAMP_26550	10089.9	12.00	10.79	12.86	1.11
15	dbAMP_26551	10380.2	11.00	10.68	11.932	1.10
16	dbAMP_26552	9920.7	12.00	10.86	12.93	1.09
17	dbAMP_26553	10227.1	14.00	11.24	15.02	0.98
18	dbAMP_26554	8892.36	11.00	11.30	11.99	0.98
19	dbAMP_26555	9504.09	11.00	10.98	12	1.06
20	dbAMP_26567	11017.2	18.00	11.96	18.99	0.99
21	dbAMP_26947	10312	9.00	10.98	10.59	0.85
22	dbAMP_26948	10477.2	11.00	11.28	12.59	0.92
23	dbAMP_26949	10451.2	11.00	11.43	12.59	0.85
24	dbAMP_26950	10375.2	15.00	11.44	16.49	0.98
25	dbAMP_32554	2680.2	3.00	10.97	3	0.39
26	dbAMP_34428	2102.29	2.00	11.23	2	0.74

### Cytotoxic effect of RD-NVs on RAW264.7 macrophages

**Figure 5.**
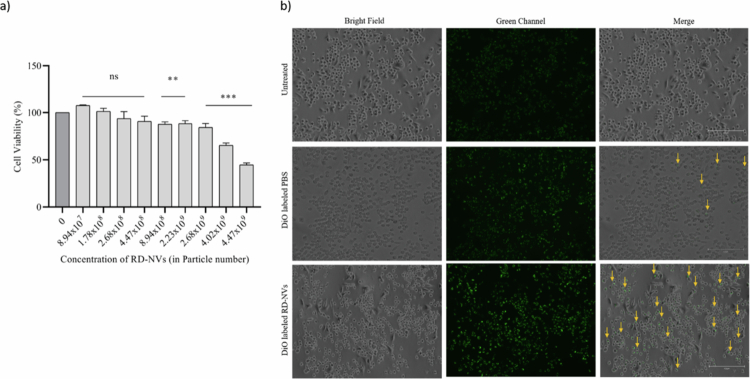
Cytotoxicity and uptake of RD-NVs in RAW264.7 macrophages. (a) Cytotoxic effects of RD-NVs on RAW 264.7 macrophages. Cells were treated with varying concentrations of RD-NVs (0 (control) 0, 8.94 × 10^7^, 1.78 × 10^8^, 2.68 × 10^8^, 4.47 × 10^8^, 8.94 × 10^8^, 2.23 × 10^9^, 2.68 × 10^9^, 4.02 × 10^9^, 4.47 × 10^9^ particles/mL) for 24 h, and cell viability was assessed to evaluate biocompatibility. Statistical significance was denoted as ***(*p *< 0.001),**(*p *< 0.01), and ns,termed as not significant in comparison with the untreated control. The F-statistic is reported as F(9,20) = 74.85 (b) Uptake of DiO-labeled RD-NVs in RAW 264.7 macrophages after 30 min incubation, visualized in RD-NVs treated, DiO- labeled PBS, and untreated cells (without RD-NVs) (scale bar: 150 μm 20× magnification).

The cytotoxic effect of RD-NVs was assessed using RAW264.7 cells exposed to varying concentrations of RD-NVs (0, 8.94 × 10^7^, 1.78 × 10^8^, 2.68 × 10^8^, 4.47 × 10^8^, 8.94 × 10^8^, 2.23 × 10^9^, 2.68 × 10^9^, 4.02 × 10^9^, or 4.47 × 10^9^) (Figure 5a). After 24 h of treatment, cell viability was determined by MTT assay. The result indicated that RD-NVs exhibited negligible cytotoxicity up to 8.94 × 10^8^ particles compared to the untreated control group. In contrast, RD-NVs administered at higher doses of 4.47 × 10^9^ particles resulted in a marked reduction in cell viability, as indicated by a decrease of less than 50% shows the higher concentration might lead to deplete the cell viability. Hence, the study chose the non-toxic range of (4.47 × 10^8^ particles) for further analysis.

### RD-NVs uptake by RAW264.7 macrophages was observed using fluorescent microscopy

RAW264.7 macrophages were incubated with or without RD-NVs (4.47 × 10^8^ particles) for 30 min. The greater uptake of RD-NVs was observed in RD-NV-treated cells compared to DiOlabeled PBS treated group (Figure 5b). The prominent green fluorescence signals in the cytoplasm confirmed the significant internalization of RD-NVs by RAW264.7 macrophages. (EVOS M5000 Imaging System; scale bar: 150 μm).

## Discussion

The morphological and biophysical features confirmed the isolation of intact nanovesicles from *R. damascena*. The spherical vesicle morphology and size range observed in TEM fall within the established dimensions for PD-NV, which generally range from 50 to 500 nm. The narrow distribution captured by NTA further indicates that the isolation method yielded a relatively homogeneous population, minimizing contamination from cellular debris or larger macromolecular aggregates.

The size distribution of RD-NVs showed variation depending on the analytical technique employed. TEM analysis revealed vesicle size predominantly in the range of 40–100 nm, representing dehydrated vesicular structures visualized under vacuum conditions. In contrast, fluorescent NTA analysis demonstrated a unimodal size distribution with a median diameter (X50) of 297.5 nm and a mean diameter of 305 nm. Such differences between electron microscopy and NTA are commonly reported and arise from fundamental methodological differences. TEM measures vesicle size in a dehydrated state under vacuum conditions, often leading to apparent size reduction and preferential visualization of smaller vesicles. In contrast, NTA measures hydrodynamic diameter in suspension, reflecting the vesicle together with its associated hydration shell and surface-bound biomolecules. Importantly, the NTA profile demonstrated a unimodal distribution rather than a multimodal or broad heterogeneous pattern, suggesting the absence of significant aggregation. The relatively narrow full width at half maximum (FWHM; 28.6 nm) further supports a concentrated size population. However, fluorescent labeling (DiO) may slightly increase the measured hydrodynamic diameter due to membrane incorporation of the lipophilic dye. Additionally, fluorescent NTA preferentially detects larger and more strongly scattering particles, potentially underrepresenting smaller vesicles (<70–100 nm), which may partly explain the shift relative to TEM observations. Similar discrepancies between imaging-based and hydrodynamic techniques have been reported in microbial and plant vesicle studies, particularly by Dean et al., where smaller vesicles detected by microscopy fall below the optimal detection threshold of nanoparticle tracking instrumentation. Therefore, the observed size variation likely reflects methodological differences combined with intrinsic vesicle heterogeneity rather than significant aggregation or large contaminating particles.^21^

The moderately negative zeta potential (−27 to −40 mV) reinforces the stability of the vesicle suspension. Plant NVs typically exhibit negatively charged membranes due to the presence of phospholipids, glycoproteins, and acidic polysaccharides, which support membrane integrity and facilitate long-distance transport in biological systems.^22^ The zeta potential values in this study, therefore, align well with reported characteristics of vesicles from edible and medicinal plants, indicating that the RD-NVs are structurally stable and suitable for downstream biological assays.^23^

The SDS-PAGE profile (Figure 1d) further confirms that the isolated vesicles contain a diverse array of proteins, including mid-range molecular weight species commonly associated with metabolic activity, stress responses, and vesicle biogenesis. The presence of multiple distinct protein bands is consistent with known plant NV cargo, which often includes enzymes, transport proteins, and stress–response regulators. This protein complexity provides a strong foundation for functional proteomics and supports the biological significance of the vesicle preparations.

Collectively, the physical, biochemical, and structural attributes observed here support the successful enrichment of RD-NVs and establish a reliable platform for subsequent molecular characterization, pathway analysis, and exploration of potential cross-kingdom biological effects. These foundational results justify the downstream investigation of RD-NV protein cargo and its implications for human cellular pathways, as detailed in the following sections.

A central finding of this study is the discovery that RD-NVs encapsulate a tightly organized chloroplast functional module rather than fragmented plastid debris. The enrichment of proteins regulating photosynthetic electron transfer (psbA, psbD, psbC, psbH) and plastid protein-folding and proteostasis (CLPP4) indicates that the NVs selectively carry metabolically active, stress-responsive enzymatic systems. Chloroplast redox regulators such as rbcL, accD, rpoA, and rpoC1 naturally operate under high oxidative load, and their presence in NVs implies a pre-adapted antioxidant architecture capable of functioning in diverse biological environments. This finding establishes a mechanistic rationale for the well-documented antioxidant and therapeutic properties of *R. damascena*, newly elucidated its beneficial outcomes at the vesicle proteome level.

Several studies on edible PD-NVs such as ginger, citrus, and grape have demonstrated antioxidant, wound healing, and mitochondrial-protective effects in mammalian systems.^19,24,25^ These reports align with the metabolic and redox-associated proteins identified in *R. damascena* NVs, suggesting that their bioactivity may follow conserved cross-kingdom mechanisms. Notably, citrus and aloe NVs promote epithelial repair, supporting the potential relevance of the proline- and retinoid-associated pathways.^19,26^ Furthermore, the celery NVs reported that the enriched proteins identified were relatively associated to protein biogenesis, degradation, and proteosome and phagosome signaling pathway.^12^

Perhaps the most scientifically intriguing observation is the functional alignment between RD*-*NV proteins and human mitochondrial pathways. The RD-NV proteome mapped robustly to mitochondrial ribosomal proteins (MRPS2, MRPS7, MRPS11, MRPS12) and ATP synthase components (ATP5F1A, ATP5F1B, ATP6V1A/B1/B2), as well as enzymes governing nitrogen and proline cycling (OAT, ALDH18A1, PYCR1) this is unlikely to be coincidental. Mitochondria and chloroplasts share an evolutionary bacterial ancestry, and our findings suggest that RD-NV cargo retains structural or functional domains that permit interaction with analogous human biochemical modules. This implies a potential cross-kingdom metabolic communication mechanism, wherein RD-NV proteins could influence mammalian mitochondrial activity, a novel concept with profound implications for nanovesicle therapeutics, nutrition science, and xenobiology. RD-NVs appear to contain an entire suite of mitochondrial-intersecting proteins, revealing a broad, multi-enzyme metabolic interface.

In addition to these primary metabolic signatures, further inspection of the proteomic dataset revealed several other conserved components with strong implications for human physiology. The NVs cargo contained multiple ribosomal proteins (including MRPS2, MRPS7, MRPS11, MRPS12, RPS14, RPS15, and RPL23), suggesting a potential role in supporting translational stability and cellular recovery during metabolic stress. The enrichment of ATP synthase and V-ATPase subunits (ATP5F1A, ATP5F1B, ATP6V1A, ATP6V1B1, ATP6V1B2) indicates that *R. damascena* NVs may interface with human mitochondrial bioenergetic pathways, potentially enhancing proton-coupled ATP production and lysosomal acidification processes central to autophagy, neuroprotection, and cellular homeostasis. The presence of enzymes mapping to vitamin and neurotransmitter biosynthetic pathways, including PDXDC1, GAD1, GAD2, DDC, and CSAD, highlights possible cross-kingdom contributions to retinoid signaling, vitamin B6 metabolism, GABAergic activity, and homocysteine clearance. Moreover, the identification of the plastid protease CLPP4, which aligns functionally with the human mitochondrial protease CLPP, suggests a conserved proteostasis mechanism capable of modulating mitochondrial protein quality control. Together, these additional molecular signatures broaden the potential therapeutic landscape of RD-NVs, linking their proteome not only to antioxidant and metabolic pathways but also to cellular regeneration, neuroprotection, and mitochondrial resilience in human systems.

The identification of RD-NV proteins mapping to human carotenoid-cleaving dioxygenases such as BCO1 and PLP-dependent amino acid decarboxylases (GAD1, GAD2, DDC, CSAD) introduces a compelling mechanistic dimension. These pathways regulate retinoid-controlled epithelial repair and immunomodulation, neurotransmitter synthesis, homocysteine detoxification, and oxidative stress response. Such pathways are rarely observed together in PD-NV studies, thus marking *R. damascena* as a uniquely bioactive vesicle source with potential roles in neuroprotection, anti-inflammatory signaling, and skin repair. The arginine‒ornithine‒proline pathway is deeply conserved and central to collagen production, mitochondrial redox regulation, osmotic protection, and regulation of nitric oxide balance. The fact that RD-NV proteins strongly map to this axis, including ARG1, ARG2, and PYCR1, suggests the vesicles may support tissue regeneration and antioxidant defense in mammalian systems. This provides biological credibility to traditional uses of *R. damascena* in wound healing and skin health.^27^ Taken together, the data support a cohesive mechanistic interpretation: RD-NVs carry a plastid-origin redox and proteostasis module that is structurally compatible with human mitochondrial bioenergetic and amino-acid metabolic systems, enabling potential cross-kingdom modulation of oxidative stress, energy production, including ATP5F1A-linked ATP synthesis, and tissue repair pathways. The integration of plastid-derived regulators (psbA, rbcL, CLPP4) with human metabolic orthologs (BCO1, PYCR1, ATP5F1A) provides a biologically plausible pathway through which RD-NVs may exert therapeutic benefits.

In advance, the peptidomic analysis of *R. damascena* NV protein cargo revealed an additional functional layer by uncovering multiple bioactive peptides with high similarity to known AMPs. From the total NV-derived peptide pool, twenty-six peptides showed >90% sequence similarity against the curated dbAMP3 database, suggesting a strong enrichment of conserved AMP-like motifs in the vesicle proteome. Notably, a subset of these peptides shared high homology with plant-derived ribosomal and small basic antifungal peptides described in the U.S. patent US6573361B1, wherein FCWP family peptides demonstrated broad-spectrum antifungal efficacy against agriculturally relevant pathogens, including *Fusarium culmorum, Botrytis cinerea, Alternaria brassicicola, and Rhizoctonia solani* (Patent US6573361B1). The convergence of peptide signatures identified in the present dataset with those experimentally validated antifungal sequences suggests that RD-NV cargo is not only metabolically conserved but may additionally contain plant defense-associated polypeptides capable of modulating plant-microbe interactions.

Interestingly, a peptide in the RD-NV matched to an AMP previously identified in *Capsicum* spp., consistent with the presence of plant defense-associated peptides across edible species. Plant AMPs such as thionins, defensins, and ribosomal protein-derived peptides are widely recognized for their basic pI, small molecular size, and high antifungal potency Broekaert et al. (1997),^28^ and similar physicochemical characteristics were reflected in several NV-matched peptides. This observation aligns with growing evidence that plants generate short bioactive peptides through proteolytic processing of stress-related proteins, which can be selectively packaged into vesicles for export.

These results complement the proteomic findings described earlier, wherein chloroplast-encoded components dominated the protein-level cargo. Together, these data suggest that RD-NVs harbor a dual functional repertoire composed of metabolic enzymes and defense-oriented peptides. The presence of AMP-like sequences in dietary plant vesicles raises the intriguing possibility of cross-kingdom protective effects, given emerging literature supporting that plant NVs can interface not only with mammalian immune responses but also with microbial communities. While the biological role of these peptides within mammalian contexts remains unexplored, their identification provides a foundation for future screening of RD-NVs-derived AMPs in human pathogens, oral microbiome systems, and plant-pathogen models. This study is based on proteomic and bioinformatic inference and does not include functional assays to validate mitochondrial or antimicrobial effects in mammalian or microbial systems. While cross-kingdom alignment is suggested, further *in vitro* and *in vivo* studies are required to confirm mechanistic effects.

In the present study, RD-NVs were isolated from whole tissue homogenates rather than from strictly purified apoplastic extracellular vesicles. Proteomic profiling revealed the presence of proteins associated with multiple cellular compartments, including chloroplasts and mitochondria, in addition to vesicle-associated proteins. Therefore, the possibility of co-isolated intracellular components cannot be completely excluded. Although morphological and physicochemical characterization support the presence of nanovesicular structures, definitive discrimination between extracellular vesicles (EVs) and other subcellular membrane fragments in plant systems remains challenging. Well-established and universally accepted EV-enriched and negative marker panels for plants are still under development. Consequently, immunoblot validation using specific plant EV markers was not performed in this study. These considerations highlight the heterogeneous nature of plant-derived nanovesicle preparations and should be taken into account when interpreting the functional implications of the RD-NV proteome. The RAW264.7 macrophage assay confirmed that RD-NVs are nontoxic within physiological ranges and are efficiently internalized within 30 min, which is consistent with previous studies of edible NVs. Macrophages are central integrators of mitochondrial and metabolic signaling; thus, the observed cross-kingdom proteomic alignment to mitochondrial ribosome and ATP synthase pathways provides a mechanistic basis for their compatibility. Together, these data support that RD-NVs possess both biocompatibility and bioavailability at the cellular interface, enabling future exploration of metabolic and immunomodulatory outcomes. The presence of AMP-like peptides within RD-NV cargo invites the possibility of a dual role in antimicrobial as well as indirect immunomodulatory functions. Macrophages serve as innate immune sensors capable of responding to both AMPs and metabolic signals. Given that several NV-associated peptides mapped to plant defensin-like sequences, future studies should evaluate whether RD-NVs modulate macrophage cytokine responses or antimicrobial gene expression. This multi-dimensional interface between metabolic pathways, innate immunity, and AMPs highlights RD-NVs as potential biotherapeutics for mucosal environments such as oral, gut, or respiratory epithelia.

## Conclusion

This study provides a comprehensive proteomic characterization of *R.* *damascena-*derived nanovesicles (RD-NVs), revealing highly conserved chloroplast and mitochondria-aligned metabolic signatures. RD-NVs encapsulate photosystem components, redox regulators, and plastid proteostasis proteins, alongside striking similarities to human mitochondrial ribosomal proteins, ATP synthase subunits, and enzymes involved in amino acid metabolism. These cross-kingdom parallels spanning retinoid metabolism, proline-collagen biosynthesis, neurotransmitter production, and mitochondrial quality control suggest RD-NVs' potential to modulate human cellular energy balance, antioxidant defense, tissue repair, and neuroprotection. Notably, the presence of AMPs underscores their role in plant and human innate immunity via host‒pathogen regulation. Overall, these findings position RD-NVs as promising plant-derived bioactive nanovesicles for therapeutic applications, meriting further in-host validation.

## Data Availability

Not applicable.
